# Successful double-layer metal stents rotational ablation under 2-dimensional and 3-dimensional optical coherence tomography guidance: a case report

**DOI:** 10.1186/s12872-021-01965-z

**Published:** 2021-04-13

**Authors:** Tong Yaliang, Liu Guohui, Zhang Cheng, Du Beibei, Zhao Yanan, He Yuquan, Yang Ping

**Affiliations:** 1 Department of Cardiology , China-Japan Union Hospital of Jilin University, Xiantai Street No. 126, Changchun, Jilin China; 2 Jilin Provincial Cardiovascular Research Institute, Changchun, Jilin China; 3 Jilin Provincial Precision Medicine Key Laboratory for Cardiovascular Genetic Diagnosis, Changchun, Jilin China

**Keywords:** Calcified plaque, Stent underexpansion, Rotational atherectomy, Stent ablation, Optical coherence tomography

## Abstract

**Background:**

Stent ablation with rotational atherectomy has been considered a bail-out strategy for the treatment of severe stent underexpansion. Only a few reports have yet shown rotational ablation for double-layer metal struts.

**Case presentation:**

We present a case of 80-year-old female patient presented to our hospital because of worsening effort angina. Coronary angiography revealed severe in-stent restenosis in the proximal left anterior descending artery. Optical coherence tomography (OCT) examinations found that severe stenosis occurred at the overlap region with 2-layer underexpanded stents and circumferential calcification beneath them. Under the guidance of 2-dimensional (2D) and 3-dimensional (3D) OCT, we successfully performed percutaneous coronary intervention (PCI) of this lesion after adequate stent ablation, high-pressure balloon dilatation, and subsequent everolimus-eluting stent implantation. The patient recovered well uneventfully and discharged from hospital 7 days later. No restenosis occurred after 12 months.

**Conclusions:**

We report a very rare case of in-stent restenosis due to double-layer underexpanded stents. The entire percutaneous coronary intervention procedure was performed step by step under the guidance of high-resolution OCT. Our findings highlight the specific value of 2D and 3D OCT guidance in double-layer stents rotational ablation.

**Supplementary Information:**

The online version contains supplementary material available at 10.1186/s12872-021-01965-z.

## Background

Stent underexpansion, usually caused by inadequate lesion preparation in severely calcified coronary lesions, is strongly associated with long-term adverse clinical outcomes, including in-stent restenosis (ISR) and stent thrombosis [1, 2]. Stent ablation (SA) with rotational atherectomy (RA) guided by intravascular imaging has been considered a bail-out strategy for the treatment of severe focal stent underexpansion associated with excellent periprocedural and in-hospital outcomes [3]. However, there are only a few reports have yet shown an SA procedure for double-layer metal struts under both 2-dimensional (2D) and 3-dimensional (3D) optical coherence tomography (OCT) guidance.

## Case presentation

An 80-year-old woman without known cardiovascular atherosclerotic risk factors was admitted to our hospital due to worsening effort angina over 1 month. The patient had a history of PCI of the left anterior descending artery (LAD) with overlapping 2.5/24 mm and 2.75/24 mm EXCEL sirolimus‐eluting steel stents (JW Medical System, Weihai, China) due to non-ST-segment myocardial infarction (NSTEMI) at another hospital 2 years prior. Electrocardiogram (ECG) revealed ST segment depression and T wave inversion in leads V1-V4. Echocardiography showed marked hypokinesis in the anterior wall of the left ventricle, whereas the serum cardiac troponin I level increased to 1.10 ng/ml (normal range 0.0–0.04) and the creatinine kinase-MB level increased to 23.6 U/L (normal range 0–16). According to the data presented above, she was diagnosed with and managed for non-ST-segment myocardial infarction (NSTEMI).

**Fig. 1:**
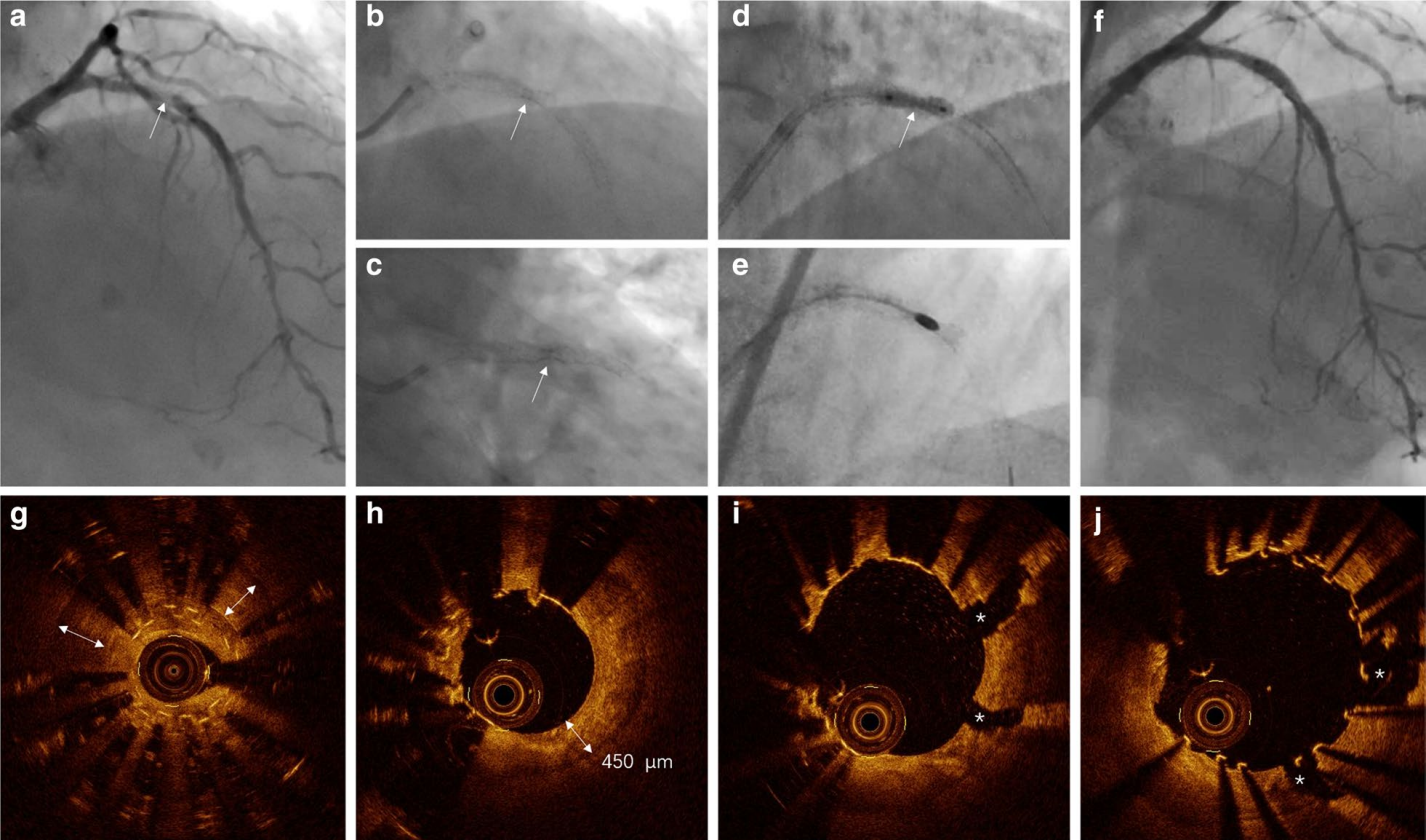
**a** Baseline coronary angiogram. **b–c** Patent stent under-expansion at the proximal left anterior descending (LAD). **d** Failed dilation using a high-pressure balloon. **e** Stent ablation with rotational atherectomy. **f** Successful result post-procedure. **g** The pre-atherectomy OCT showed severe calcification beneath the struts (white double-headed arrow). **h** The post-atherectomy OCT showed thick calcification (white double-headed arrow) after stent ablation. **i** Two clear calcium fractures (asterisk) were observed post high-pressure balloon dilation after stent ablation. **j** Final OCT post PCI

The patient received loading doses of aspirin (300 mg, p.o.), ticagrelor (180 mg, p.o.), and heparin (6000 IU, i.v.) before the PCI procedure. Coronary angiography revealed mild-to-moderate stenosis in the left circumflex coronary artery (LCX) and right coronary artery (RCA) and severe ISR in the proximal LAD (Fig. 1A). The underexpanded segment of the patent stent was observed clearly before contrast injection without cardiac motion (Fig. 1B and C). Inflation of a 2.75/12 mm noncompliant balloon (NCB, Boston Scientific Corp., Natick, MA) at high pressure (20–24 atmospheres) several times failed to dilate the resistant lesion (Fig. 1D). Then, OCT examinations were performed on a workhorse wire (Runthrough NS, Terumo, Japan) using the C7-XR OCT intravascular imaging system (LightLab Imaging Inc., St. Jude Medical, USA). The cross-sectional OCT images found that severe stenosis occurred at the overlap region with 2-layer underexpanded stents and severe calcification beneath the struts (Fig. 1G and Additional file 1). The minimal stent diameter (MSD), minimal lumen diameter (MLD), and minimal lumen area (MLA) of the lesion were 1.21 mm, 0.93 mm, and 0.68 mm^2^ by OCT, respectively. Longitudinal OCT analysis showed that the length of the overlap region was approximately 5 mm.

In order to avoid the passage of the wires through the struts and their subsequent crush, we exchanged the rota-wire through a micro-catheter (Finecross, Terumo). Double-layer metallic stents and calcification ablation were performed by rotational atherectomy (Fig. 1E) using a 1.5 mm burr (RotaLink, Boston Scientific) via a 7Fr EBU3.5 guiding catheter. The ablation speeds were between 140,000 and 150,000 rpm, and runs were kept short (≤ 15 s), with a “pecking” motion and conservative force to reduce the risk of no-reflow phenomenon, thermal injury and burr entrapment. Fortunately, the 1.5 mm burr passed the lesion with 10,000 rpm down from 150,000 rpm after about 25 runs, followed by another 10 runs for better modification. The total ablation time was 7 min, and the SA procedure was free of complications. Repeated OCT evaluation post-SA was performed on the Runthrough NS wire after wire exchange through the Finecross microcatheter. The OCT images demonstrated that the metallic struts of the underexpanded stents had completely disappeared in approximately two quadrants (Fig. 1H and Additional file 2). The length of the ablated stent portion was about 3 mm, and the minimum calcium thickness at the MLA site was 450 µm. We were confident that adequate lesion modification had been achieved and that there was no need to upsize the burr.

**Fig. 2:**
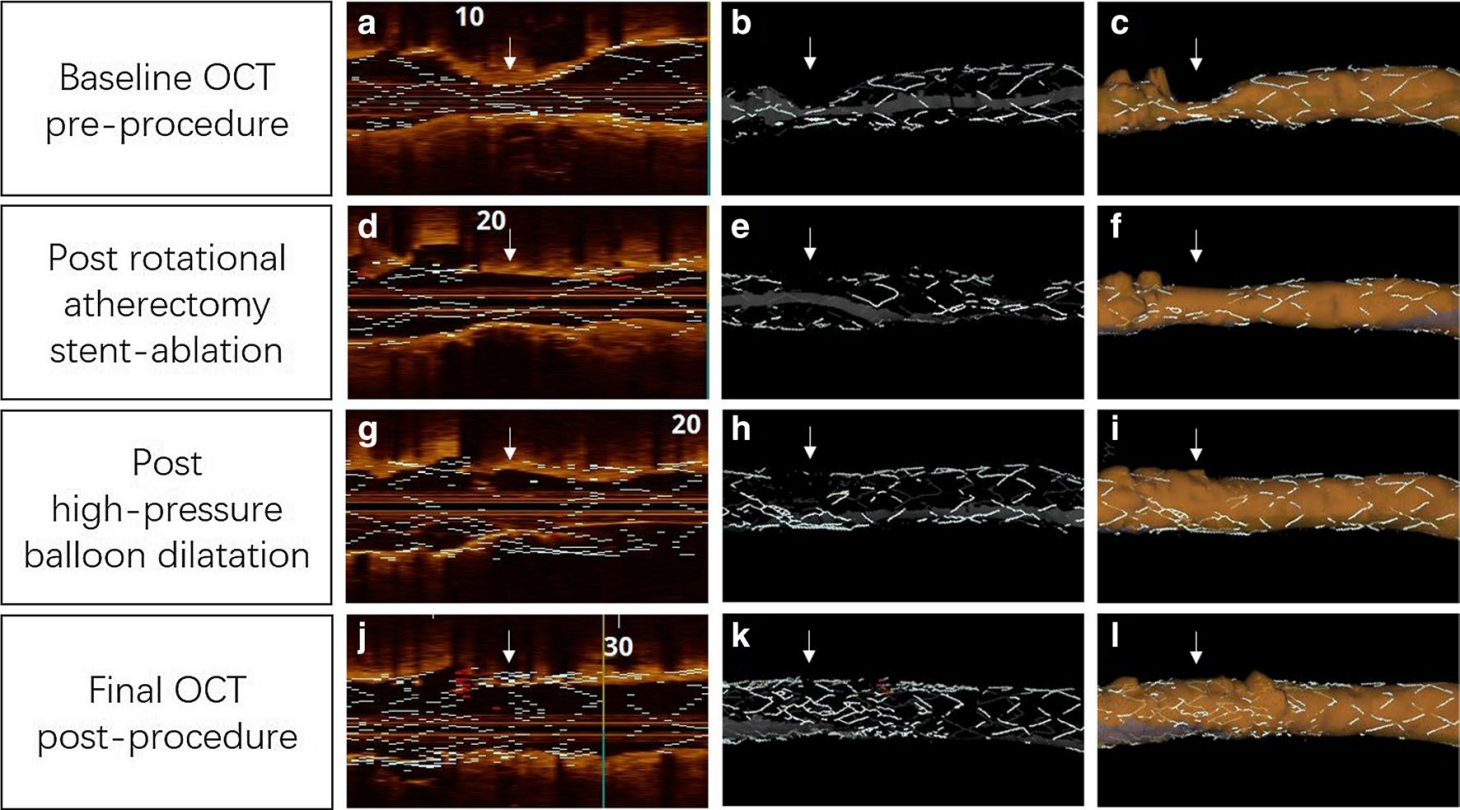
Longitudinal and 3D OCT views. **a, d, g** and **j** Tissue and lumen display mode. **b, e, h **and **k** Stent only display mode. **c, f, i** and **l** Lumen display mode. **a–c** The baseline OCT pre-procedure showed severe stenosis with stent underexpansion (white arrows). **d–f** The OCT post-stent ablation demonstrated that partial metallic struts disappeared (white arrows). **g–i** The OCT post high-pressure balloon dilatation following stent ablation showed that the target lesion has been well dilated. **j–l** The final OCT post-procedure demonstrated that the new implanted stent was well expanded

Based on the information acquired from the post-RA OCT, subsequent high-pressure dilatation was performed with a 2.75/12 mm noncompliant balloon (20 atmospheres), resulting in optimal expansion. Two clear calcium fractures were observed on the cross-section OCT images post-SA and NCB dilatation (Fig. 1I and Additional file 3). The new implanted stent size was also determined under the guidance of OCT. Both the proximal and distal landing zones were selected at the adjacent relatively normal regions with thin and intact neointima. The mean lumen diameters of the proximal and distal reference were 2.41 mm and 2.61 mm, while the mean patent stent diameters of the proximal and distal landing zones were 2.82 mm and 2.96 mm, and the length of the target lesion was about 10.5 mm (Additional file 4). Finally, we implanted a 2.75/12 mm Nano (Lepu Medical Technology, Beijing, China) polymer-free sirolimus-eluting stent (SES) that was expanded at 14 atmospheres over 30 s. After a final post-dilatation (2.75/12 mm NCB) at 24 atmospheres, a good angiographic result was achieved (Fig. 1F). The final OCT showed satisfactory apposition and expansion of the stents (Fig. 1J and Additional file 5), with an MLA = 5.53 mm^2^. Off-line analysis of the OCT images was performed using the ILUMIEN OPTIS OCT imaging system (St. Jude Medical, Minneapolis, MN, USA). Longitudinal and 3D reconstruction of the OCT images clearly showed the effect of the entire PCI procedure step by step (Fig. 2).

The patient suffered from severe shortness of breath post PCI with complete symptom relief after intravenous aminophylline 125 mg. We speculated that the transient dyspnea might be the side effect of ticagrelor. So we adjusted her DAPT strategy. The first loading doses of clopidogrel (300 mg) was administered 12 h after the ticagrelor administration. Then the patient was treated with aspirin (100 mg), clopidogrel (75 mg) once per day. She had an uneventful recovery and was discharged 7 days later on aspirin, clopidogrel, a beta-blocker, a statin, and an angiotensin receptor. Routine follow-up coronary angiography was repeated 12 months later, and the final outcome was excellent.

## Discussion and conclusion

Severe focal stent underexpansion is a known complex condition that currently occurs during repeated revascularization. Although excimer laser coronary atherectomy (ELCA) [4–7] and intravascular lithotripsy (IVL) [8] have been reported to be effective in the management of underexpanded and undilatable coronary stents, SA with RA is the only available interventional remedy to deal with intractable circumstances in most heart centers. In the past two decades, about 80 cases undergoing SA with RA have been published with excellent outcomes and minimal periprocedural complications [3]. However, there are only a few reports regarding SA for double-layer [9] or triple-layer [10] underexpanded stents using 2 burrs. In our case, successful SA was performed using only one 1.5 mm burr without upsizing. The keys for this successful procedure were slower ablation speeds (140,000–150,000 rpm), shorter runs (≤ 15 s), pecking motion, and conservative advancing force. More importantly, the information provided by high-resolution OCT allowed us to make precise decisions, including burr selection, transition strategy from SA to high pressure dilation, and optimizing stent implantation.

Recent data have demonstrated that intravascular imaging can provide additional information regarding lesion preparation, which results in higher rates of procedural success and better long-term outcomes, especially in complex lesions [3, 11]. In most published cases of SA, intravascular ultrasound (IVUS) is the most common imaging modality utilized pre- and post-RA [3]. However, it is difficult to show the efficacy of RA in severely calcified plaques together with underexpanded stents in view of IVUS because ultrasound cannot penetrate the calcification and metallic struts. OCT, with approximately 10 times better axial resolution than IVUS, can precisely detect the struts and detect the thickness of the calcified layer since near infrared light has the ability to penetrate calcium tissue without shadow artifacts. Thus, we chose to perform the entire procedure for this intractable double-layer stent underexpansion under the guidance of high-resolution OCT.

As demonstrated in our case, OCT images can provide valuable information during the entire PCI procedure: (1) Prior to stent ablation, OCT can provide a better understanding of the lesion’s detailed characteristics, including the MLA, MLD, MSA, and the presence of heavily circumferential calcification beneath the double-layer struts, which is useful for burr size selection. (2) Post-RA OCT can help in the process of making further therapeutic decisions. When the metallic struts completely disappeared in approximately two quadrants and the minimum calcium thickness at the MLA site was less than 450 µm, we were confident that adequate lesion modification was achieved. Thus, we prefer to perform high-pressure balloon dilatation instead of continuing ablation or unnecessarily upsizing the burr. (3) As noted previously, OCT has the ability to precisely detect calcified tissue. Therefore, we can observe calcium fractures clearly post high pressure balloon dilation, ensuring perfect lesion preparation. (4) Finally, under the guidance of subsequent OCT, we can optimize stent implantation with an appropriately sized stent, landing zones and postdilation. (5) Additionally, high-resolution OCT enables longitudinal and 3D reconstruction of the target lesion and permits direct steep visualization of the entire PCI procedure.

In conclusion, stent ablation for the management of 2-layer underexpanded stents is a viable technique of which operators should be aware. Intravascular imaging can provide additional information regarding precise strategy of repeated PCI. To our knowledge, this is the first detailed analysis of the guiding value of 2D and 3D OCT in double-layer stents rotational ablation step by step.

## Supplementary Information

**Additional file 1.** Baseline OCT pre-procedure.

**Additional file 2.** OCT post-stent ablation.

**Additional file 3.** OCT post high-pressure balloon dilatation following stent ablation (AVI 6.2 MB)

**Additional file 4.** The proximal and distal landing zones of the new implanted stent. **a** The distal landing zone. **b** The target lesion. **c** The proximal landing zone. **d** The longitudinal OCT view showed the length of the target lesion.

**Additional file 5.** Final OCT post-procedure.

## Data Availability

All data generated or analyzed during this study are included in this published article.

## Abbreviations

OCT: Optical coherence tomography
PCI: Percutaneous coronary intervention
ISR: In-stent restenosis
SA: Stent ablation
RA: Rotational atherectomy
LAD: Left anterior descending artery
ECG: Electrocardiogram
NSTEMI: Non-ST-segment myocardial infarction
LCX: Left circumflex coronary artery
RCA: Right coronary artery
NCB: Noncompliant balloon
MSD: Minimal stent diameter
MLD: Minimal lumen diameter
MLA: Minimal lumen area
ELCA: Excimer laser coronary atherectomy
IVL: Intravascular lithotripsy
IVUS: Intravascular ultrasound
