# Supplementary figures and images for: Successful double-layer metal stents rotational ablation under 2-dimensional and 3-dimensional optical coherence tomography guidance: a case report
Source: BMC Cardiovasc Disord. 2021 Apr 13;21:173. doi: 10.1186/s12872-021-01965-z (PMC8042855; doi:10.1186/s12872-021-01965-z)

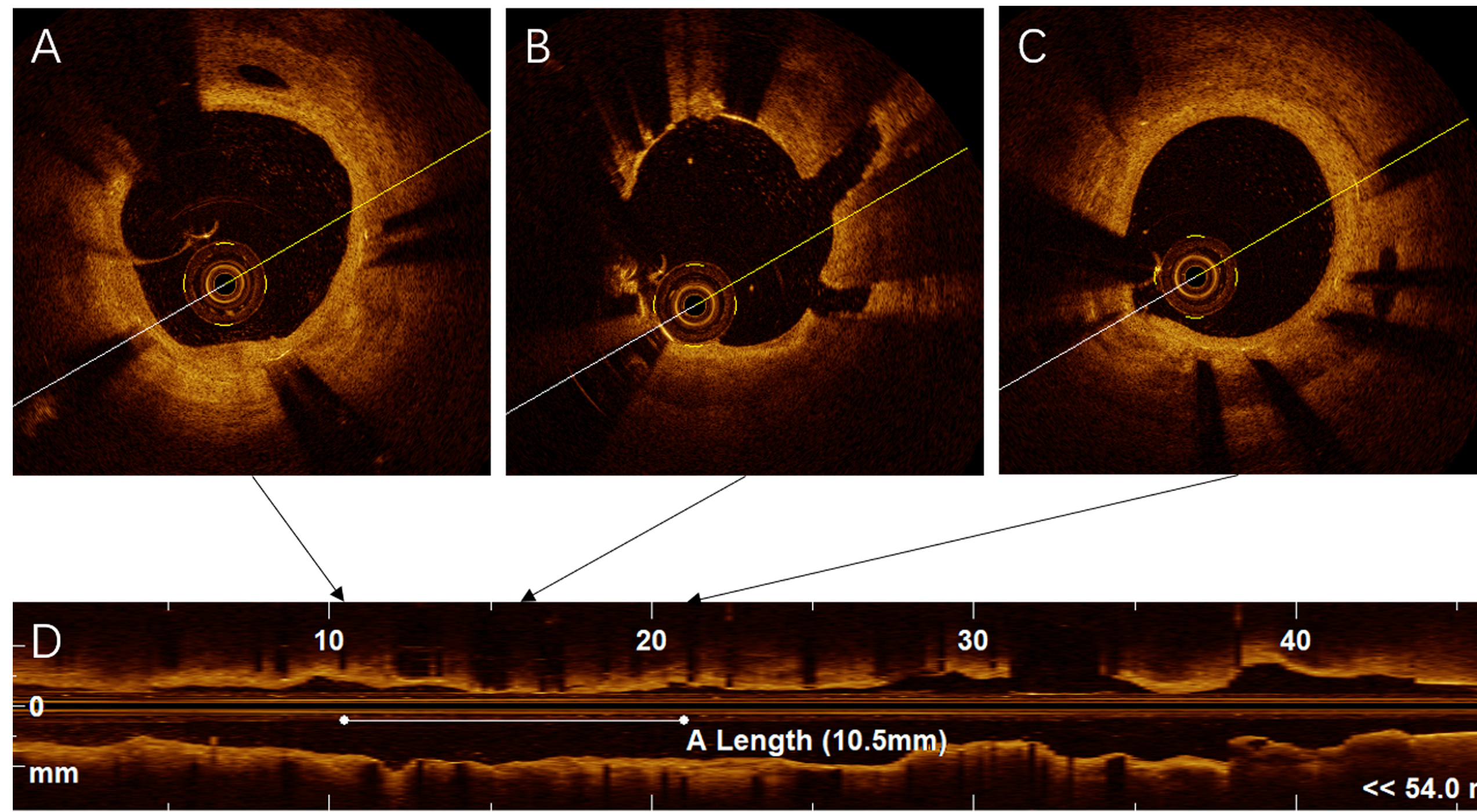

Supplement: Supplementary file 4 — Additional file 4. The proximal and distal landing zones of the new implanted stent. a The distal landing zone. b The target lesion. c The proximal landing zone. d The longitudinal OCT view showed the length of the target lesion. [file 12872_2021_1965_MOESM4_ESM.jpg]
